# Protein Kinase C-epsilon in Membrane Delivery during Phagocytosis

**DOI:** 10.29245/2578-3009/2018/2.1134

**Published:** 2018-04-18

**Authors:** Anna E. D’Amico, Michelle R. Lennartz

**Affiliations:** 1 Department of Regenerative and Cancer Cell Biology, Albany Medical College, 47 New Scotland Avenue Albany, NY 12208, USA

**Keywords:** Macrophages, Protein kinase C-epsilon, Phagocytosis, Phosphatidylinositol-4-phosphate, Trans Golgi Network, Vesicle scission

## Abstract

During phagocytosis, internal membranes are recruited to the site of pathogen binding and fuse with the plasma membrane, providing the membrane needed for pseudopod extension and target uptake. The mechanism by which vesicles destined for the phagosome are generated, targeted, and fuse is unknown. We established that Golgi-associated protein kinase C-epsilon (PKC-ε) is necessary for the addition of membrane during FcyR-mediated phagocytosis. PKC-ε is tethered to the Golgi through interactions between its’ regulatory domain and the Golgi lipids PI4P and diacylglycerol; disruption of these interactions prevents PKC-ε concentration at phagosomes and decreases phagocytosis. The accumulated evidence suggests that PKC-ε orchestrates vesicle formation at the Golgi by a mechanism requiring lipid binding but not enzymatic activity. This review discusses how PKC-ε might mediate vesicle formation at the level of budding and fission. Specifically, we discuss PKC-ε binding partners, the formation of lipid subdomains to generate membrane curvature, and PKC-ε mediated links to the actin and microtubule cytoskeleton to provide tension for vesicle fission. Assimilating information from several model systems, we propose a model for PKC-ε mediated vesicle formation for exocytosis during phagocytosis that may be applicable to other processes that require directed membrane delivery and fusion.

## Introduction

Our recent papers^1,2^ provide insight into the focal exocytosis that underpins pseudopod extension during Fcγ receptor (FcγR)-mediated phagocytosis. We demonstrate that the pseudosubstrate of protein kinase C-epsilon (PKC-ε) tethers PKC-ε to the Golgi by binding phosphatidylinositol -4- phosphate (PI4P). Deletion of the pseudosubstrate, or removal of Golgi PI4P, prevents PKC-ε translocation to forming phagosomes and the membrane fusion required for pseudopod extension. The novelty of these findings lies in the discovery that the pseudosubstrate, previously thought to function only to keep PKC inactive, binds lipids and plays an essential role in the localization and translocation of a PKC in response to receptor ligation. This is the first example of a PKC that translocates to the plasma membrane on a vesicle rather than from the cytosol.

## Background

**Figure 1.**
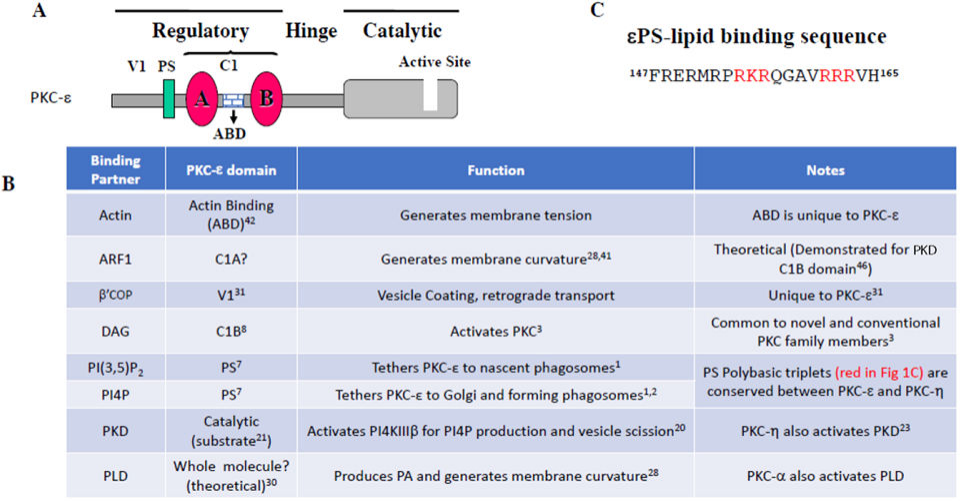
(A) Domain structure of PKC-ε. (B) Table listing the binding location and function of proteins that interact with PKC-ε. (C) Sequence within the pseudosubstrate region of PKC-ε required for translocation; polybasic triplets are highlighted in red. See text for details.

Structurally, PKCs have a homologous catalytic domain connected to a variable regulatory domain by a flexible hinge (Figure 1A). The superfamily contains 10 isoforms: classical, novel, and atypical, classified based on their activators^3^. Mature PKCs are predominantly cytosolic, held in a closed conformation by the presence of the pseudosubstrate in the active site. Upon cell stimulation, generation of PKC activators (e.g., diacylglycerol, rise in calcium, accessibility of protein binding partners)^3^ promote PKC’s translocation to the plasma membrane where it undergoes a conformational change that releases the pseudosubstrate, focally activating the enzyme. This mechanism is well documented for the classical PKCs^4^. Our work with PKC-ε suggests that translocation of PKC-ε is different^1, 2^.

**Figure 2.**
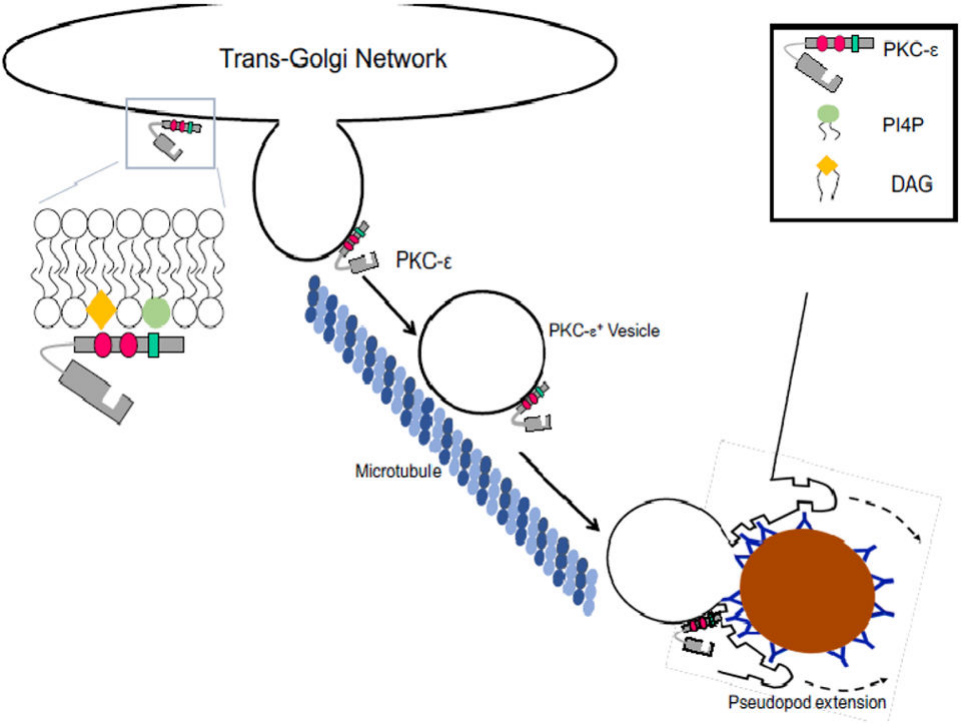
Overview of TGN-to-phagosome vesicular trafficking. PKC-ε is tethered to the TGN through DAG-εC1B and εPS-PI4P interactions. PKC-ε^+^ vesicles travel on microtubules to the plasma membrane beneath bound targets. While the regulatory domain is sufficient for vesicle formation and translocation, catalytic activity is required for membrane fusion for pseudopod extension. See text for details.

PKC-ε is involved in such varied processes as cytokinesis^5^, neurotransmission^6^, neurite extension^7^, and phagocytosis^1, 8, 9^. A common feature of these processes is focal exocytosis, with fusion allowing release of vesicle contents and membrane expansion (Figure 2). Dysregulation of PKC-ε is associated with pathologies including infection^10^, defects in wound healing^11^, tumor cell proliferation/metastases^12–14^ and Alzheimer’s disease^15^. Phagocytosis provides a model for studying focal exocytosis as membrane fusion occurs selectively at sites of pathogen binding.

## The pseudosubstrate of PKC-ε is required for translocation to forming phagosomes

We previously demonstrated that PKC-ε concentrates beneath bound targets^16^ and that blocking this concentration (or its’ absence in PKC-ε null macrophages) abolishes FcγR-dependent membrane fusion, significantly reducing phagocytosis^9, 16^. As PKC-ε is activated by diacylglycerol (DAG), it was no surprise that translocation to forming phagosomes requires DAG and the (DAG binding) domain of PKC-ε, εC1B^8^ (Figure 1B). Chimeras of PKC-ε and PKC-δ (a novel PKC that does not concentrate during phagocytosis^16^) revealed that the pseudosubstrate of PKC-ε (εPS) was also required for translocation^9^. We defined a minimal chimeric fragment (amino acids 147–165 from εPS and the xC1B region) that is necessary and sufficient for concentration at phagosomes^9^. This was the first demonstration that the pseudosubstrate of any PKC plays an active role in translocation.

εPS contains two polybasic triplets (Figure 1C), a motif characteristic of phosphoinositide phosphate (PIP) binding domains^17^. Liposome binding assays identified PI4P and PI(3,5)P_2_ as εPS binding partners^9^. Studies using alanine mutants of the polybasic triplets suggest that specificity for phagosome concentration is encoded in one or more of the 154RKR156 residues^9^. This triplet is preceded by a proline that may kink the peptide, exposing the triplet to membrane PIPs.

Given that pseudopod extension is dependent on PI3 kinase^18^, that εPS binds PI(3,5)P_2_^9^, and that PKC-ε is necessary for phagocytosis^8, 16^, we predicted that inhibition of PI3 kinase would block PKC-ε translocation (and phagocytosis)^1^. While wortmannin produced the expected effect, we were surprised that the more specific PI3 kinase inhibitor, LY249002, blocked neither PKC-ε translocation nor phagocytosis^1^. As wortmannin, but not LY, inhibits type III PI4 kinases^19^ and εPS also binds PI4P, we hypothesized that PKC-ε requires PI4P for concentration at phagosomes. Support for this was based on the knowledge that PI4P is enriched in the trans Golgi network (TGN) and that PKC-ε concentrates in a perinuclear pattern^1^. Additionally, live imaging revealed that the Golgi-associated PKC-ε disappears as PKC-ε increases at the phagosome^1^. PKC-ε at the TGN can be reduced/eliminated by expressing the PI4P reporter P4M (competes with PKC-ε for PI4P binding), inhibiting the type III PI4Ks with PIK93, or depleting Golgi PI4P by expression of a Golgi-directed PI4 phosphatase^1^. The decrease in the Golgi is paralleled by reduced PKC-ε concentration at the forming phagosome and loss of FcγR-dependent membrane fusion (as quantified by whole cell patch clamping)^1^. Ongoing imaging studies suggest that PKC-ε moves to the forming phagosome on vesicles, vesicles that are not delivered in PKC-ε^−/−^ macrophages. Taken together, these data suggest that PKC-ε orchestrates the formation of vesicles at the Golgi, a model that is supported by studies demonstrating that polybasic triplets, such as those in εPS, are export signals in Golgi-to-plasma-membrane trafficking^20^.

## Vesicle Formation

Studying vesicle formation in isolated Golgi preparations has proven difficult. Thus, much of the information on vesicle formation comes from biophysical studies using artificial membranes. What follows is a theoretical analysis, based on results from many systems, of how PKC-ε could orchestrate the formation of TGN-derived vesicles that translocate to the membrane beneath bound targets, fusing into the forming phagosome to provide membrane for pseudopod extension (Figure 2).

**Figure 3.**
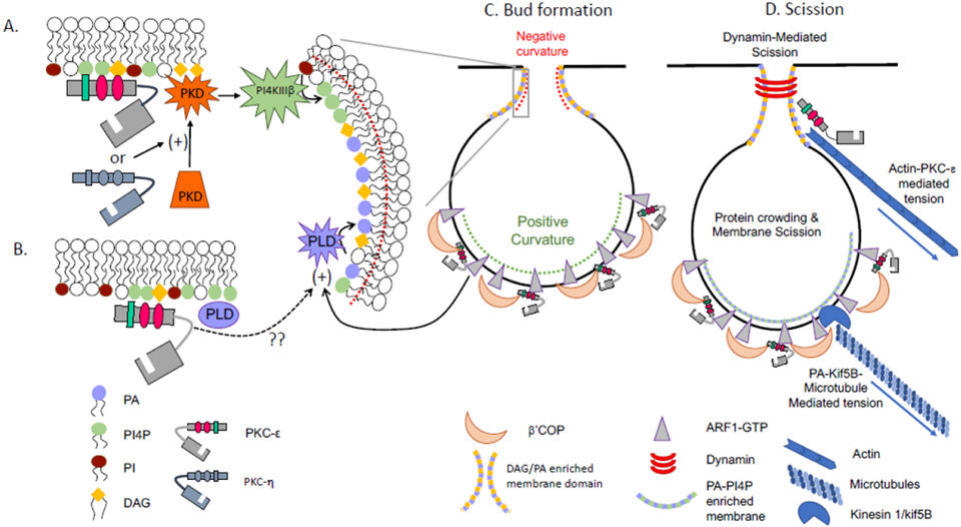
Model for PKC-ε involvement in vesicle formation at the TGN. (A) PKD binds DAG in the TGN. PKD can be activated by PKC-ε or its’ close relative PKC-η. Activated PKD stimulates PI4KIIIb to focally increase PI4P concentration, a lipid required for vesicle budding. (B) As PKC-ε action at the TGN does not require catalytic activity, and PLD is activated by a catalytically inactive PKC, the regulatory domain of PKC-ε could potentially activate PLD. PLD is also activated by the small GTPase ARF1. PKC-ε indirectly binds ARF1 through β’COP and could facilitate PLD activation through this interaction. (C) ARF1-GTP inserts into membranes generating positive curvature. Through its’ lipid binding, PKC-ε may mark preferred sites β’COP binding which, in turn, would recruit ARF1. Alternatively, there is precedence for the direct binding of ARF by C1 domains; the C1A domain in PKC-ε could theoretically bind ARF. (D) Vesicle scission could proceed by polymerization of dynamin around the neck of vesicle, generated by negative membrane curvature resulting from enrichment of DAG and PA. The radius of the neck could be decreased through tension applied by actin (attached to the vesicle through the actin binding domain of PKC-ε) and/or through kif5B-microtubule generated force, with kif5B interacting with PA to link the vesicle to the cytoskeleton. See text for details.

Vesicle formation requires membrane budding followed by scission. Budding requires generation of membrane curvature, which can occur by external forces, including assembly of protein coats or oligomerization of crescent-shaped BAR proteins on the membrane, and/or by altering lipid packing in the membrane through enrichment of wedge-shaped lipids. Lipid packing could take the form of negative member curvature such as that at the neck of vesicle buds, or positive curvature required for expansion of the bud (Figure 3C). DAG and phosphatidic acid are wedge-shaped lipids that promote negative curvature while small molecular weight GTPases, such as ARF1, insert into the membrane to generate positive curvature.

## PKC-ε and vesicle budding

PKC-ε is tethered to the trans-Golgi network (TGN) via εPS-PI4P and εC1B-DAG^1, 8, 9^. In addition to PKC-ε, DAG can tether other C1-containing proteins, including protein kinase D (PKD)^21^. PKD is required for TGN-to-plasma transport; its’ inhibition retains cargo in TGN-associated tubules, suggesting a role in vesicle scission^21^. While these studies were done in HeLa cells, PKD may function similarly during phagocytosis. PKC-ε phosphorylates PKD^22^ which, in turn, activates PI4KIIIb, causing a rise in PI4P (Figure 3A). As PI4P concentrates at sites of exocytosis and its’ loss inhibits secretion^23^, a PKC-ε→PKD→PI4KIIIb→↑PI4P pathway for PKC-ε dependent vesicle formation can be envisioned (Figure 3A).

The caveat to this model is that it relies on PKC-ε phosphorylation of PKD and we know that PKC-ε action at the Golgi does not require catalytic activity^1, 9^. However, macrophages express several PKC isoforms, including PKC-eta (PKC-η). The PS and C1B domains of PKC-η and PKC-ε are highly homologous, sharing 58% and 86% identity, respectively, including the PS polybasic triplets. PKC-η localizes to the Golgi, activates PKD, and its’ inhibition produces Golgi-tethered tubules^24^. While these studies were done in HeLa and 293T cells, macrophages could utilize a similar pathway (Figure 3A). Assuming that PKC-η activates PKD, phagocytosis and vesicle delivery are impaired in PKC-ε^−/−^ cells despite the expression of PKC-η. Clearly PKC-ε has a role beyond phosphorylation of PKD, although that role needs to be elucidated.

Like DAG, phosphatidic acid (PA) is a wedge-shaped lipid that contributes to negative membrane curvature. PA is the product of phospho**lipase** D (P**L**D) which is activated during, and required for, FcγR-mediated phagocytosis^25^. That PLD stimulates vesicle release from the TGN in GH3 cells^26^ provides precedence for a similar role in phagocytosis. While a direct link between PLD and PKC-ε has not been established, two pathways can be envisioned. PLD is activated by a Ca-independent, PMA binding PKC^27^. As this PKC effect is independent of catalytic activity, εRD could theoretically activate PLD. Secondly, PLD is activated by ARF1, which could be mediated by the εRD-ARF1 interaction (see below)^28^. By binding DAG and activating PLD, εRD would bring together DAG and PA, lipids that would induce negative membrane curvature at the neck of budding vesicles and facilitate fission (Figure 3B).

ARF1, a small molecular weight G-protein, forms tubules from PI4P-containing lipid bilayers^29^. In its’ GTP-bound form, ARF1 inserts into the lipid bilayer, generating positive membrane curvature and orchestrating assembly of COPI coats. While COPI primarily mediates retrograde Golgi trafficking, it has also been implicated in IgG-mediated phagocytosis. The data suggests that COPI acts at a step prior to phagosome formation^30^, consistent with a role in vesicle formation. That the ß’COP subunit of the COPI coat binds both ARF1^31^ and the V1 domain of PKC-ε^32^ links PKC-ε to vesicle budding by bringing together DAG (negative curvature), ARF-1 (positive curvature), and coat proteins (ßl’COP) (Figure 3C).

Due to the physical constraints on phospholipid bilayers, it is generally assumed that vesicle formation requires a coat, with COPI and clathrin being linked to TGN trafficking. The evidence for the involvement of COPI is presented above with the caveat that COPI vesicles function in retrograde transport. Similarly, while molecules involved clathrin-mediated trafficking (ie, AP-1^33^, dynamin II^34^) are implicated in phagocytosis, downregulation of clathrin does not impact the process^34^. To date, there is no direct evidence that vesicles destined for the phagosome are coated. An alternative would be the recently described CARTS (CARriers of the TGN to cell Surface pathway). While little is known about their formation, CARTS have several features^35^ in common with PKC-ε^+^ vesicles: CARTS carry selective cargo (during phagocytosis, vesicles that fuse into the phagosome carry TNF-α, but not IL-6^36^), CARTS express TGN46 (the mouse homologue TGN38 co-localizes with PKC-ε in macrophages, Lennartz, unpublished observation), and PKD is required for CARTS release from the Golgi^35^. Finally, transport of CARTS in HeLa cells utilizes Kinesin-5/Eg537; Kinesin 1 (kif5B) is required for delivery of membrane during phagocytosis in macrophages^37^. Open questions include: Are PKC-ε^+^ vesicles coated? What is the role of CARTS in PKC-ε-mediated membrane delivery for phagocytosis?

## Membrane Scission

Vesicle release from the TGN requires membrane scission. The GTPase dynamin polymerizes on membrane tubules and, upon GTP hydrolysis, constricts and torques the membrane, resulting in fission^38^. In this in vitro system, fission was independent of coat proteins but membrane tension was required^38^. While the paradigm is that dynamin binds PI(4,5)P_2_ to initiate polymerization (and there is little PI(4,5)P_2_ on TGN membranes), in vitro studies revealed that membrane curvature dictates polymerization^39^. That is, dynamin spontaneously polymerizes on tubules of 10–30 nm radius^39^. While the radius of the necks of PKC-ε^+^ vesicles is unknown, the negative curvature induced by production of DAG and PA (Figure 3) could generate a “tube” of appropriate radius to support spontaneous polymerization of dynamin. The application of tension (see below) may further decrease the radius of the neck, facilitating fission. Combined with the evidence that dynamin-GFP moves towards the phagosome and its down-regulation blocks exocytosis and pseudopod extension^40^, dynamin-mediated fission could release PKC-ε^+^ vesicles from the TGN.

Coat-independent membrane fission also occurs by “protein crowding”^41^ and “friction-driven scission (FDS)^42^. Both processes require proteins that promote membrane curvature (Figure 3). The protein crowding model reported that addition of the GTPase Sar1p to lipid coated beads is sufficient for vesicle release^41^. As Sari and ARF1 both generate membrane curvature by insertion into the bilayer^42^, ARF1 could perform a similar function in release of PKC-ε^+^ vesicles; the potential link between ARF1 and PKC-ε is detailed above.

Similarly, tubular membranes with BAR protein “scaffolds” undergo friction-driven scission (FDS) in response to microtubule-mediated membrane tension^42^. BAR (Bin-Amphiphysin-Rvs) domain proteins sense membrane curvature; when bound to a curved membrane, the BAR domain oligomerizes, propagating membrane curvature. While BAR proteins have not been linked to phagocytosis, the authors suggest that FDS is a general mechanism for scission of protein-coated membranes. Specifically, they suggest that FDS may function for scission of COPI or COPII vesicles^42^. Integrating this into what we know about phagocytosis, one could postulate that the COPI coat, rather than acting in a retrograde fashion, provides the membrane coverage necessary for FDS. COPI would provide the scaffold while ARF1 links the coat to the membrane. Tension, provided by actin or microtubules (both of which associate with the TGN), supplies the force needed for vesicle release.

With respect to the requirement for tension, PKC-ε is unique amongst the PKCs in the presence of an actin binding domain^43^. This domain, located in the C1 region, could bind actin to provide tension for vesicle formation in macrophages (Figure 3D). Microtubules are clearly involved in phagocytosis as nocodazole abolishes membrane delivery in response to FcγR ligation^1^. Additionally, the microtubule motor, kif5B is required for phagocytosis^37^. Notably, kif5B binds PA^44^, which is generated during phagocytosis by activation of PLD (see above)^24^.

## Model

In 1996, the Sabatini lab published “The Production of Post-Golgi Vesicles Requires a Protein Kinase C-like Molecule, but Not Its Phosphorylating Activity”^45^. Using purified Golgi preparations and visualizing vesicle formation by electron microscopy, the group demonstrated that Calphostin C prevented the scission of vesicles but not budding or coat assembly. By conducting the experiments in the absence of ATP, they established that vesicle release was independent of PKC activity. As Calphostin C inhibits PKC by competing with DAG for C1 binding, the authors defined their molecule as “PKC-like” and reported that it was recruited to the Golgi prior to coat assembly^45^. εRD could be this PKC-like molecule; it would bind DAG and PI4P, which would be inaccessible once a coat was assembled.

If we assume that PKC-ε is Sabatini’s “PKC-like activity” then it would be recruited before coat formation. Given that ~20% of PKC-ε is membrane associated in resting macrophages^16^, that the TGN is enriched in PI4P and DAG, and that PKC-ε-GFP concentrates perinuclearly^1, 8, 9^, PKC-ε may be constitutively TGN associated. The PLC-activation associated with FcγR ligation could increase DAG. PKD would translocate in response to the rise in DAG; PKC-η would phosphorylate and activate PKD which, in turn would increase PI4P levels by activating PI4KIIIb (Figure 3A). PKD binding of ARF1^46^ or PKC-ε would activate PLD (translocated to membranes containing anionic lipids). The PA produced would contribute to negative membrane curvature to recruit dynamin (Figure 3D), which, upon GTP hydrolysis would lead vesicle scission. Alternatively, accumulation of regions enriched in DAG and PA would cause negative membrane curvature that, with the application of tension, would promote fission. Fission could be facilitated by protein crowding and tension supplied by kif5B-microtubule and/or actin-εRD interactions. Following fission, PKC-ε^+^ vesicles would be transported on microtubules via kif5B to the phagocytic cup where PKC-ε would phosphorylate proteins involved in vesicle fusion for pseudopod extension and completion of phagocytosis (Figure 2).

As the absolute timing of phagocytosis is a function of particle size and temperature, we have reported that a glass bead of 2 μm diameter is internalized in about 80s^8^. PKC-ε concentration occurs rapidly upon target binding but is also dependent on that binding. Thus, ligated receptors must signal the Golgi to release vesicles within seconds of initial binding. What that signal is and how the vesicles are targeted exclusively to sites of FcγR ligation are questions that remain to be answered.

## Abbreviations

ABD: Actin binding domain
DAG: diacylglycerol
PKC-ε: protein kinase C-epsilon
εC1: constant region 1 of PKC-ε
εPS: pseudosubstrate region of PKC-ε
εRD: regulatory domain of PKC-ε
FcγR: Fc[gamma] receptors for IgG
PI4P: phosphatidylinositol-4-phosphate
PI4K: phosphatidylinositols-4-kinase
PKD: Protein Kinase D
PLD: Phospholipase D
TGN: Trans Golgi Network
εV1: Variable Region 1 of PKC-ε
PA: phosphatidic acid
